# Physiologic-Based Cord Clamping Maintains Core Temperature vs. Immediate Cord Clamping in Near-Term Lambs

**DOI:** 10.3389/fped.2020.584983

**Published:** 2020-10-23

**Authors:** Douglas A. Blank, Kelly J. Crossley, Aidan J. Kashyap, Ryan J. Hodges, Philip L. J. DeKoninck, Erin V. McGillick, Karyn A. Rodgers, Arjan B. te Pas, Stuart B. Hooper, Graeme R. Polglase

**Affiliations:** ^1^The Ritchie Centre, Hudson Institute of Medical Research, Clayton, VIC, Australia; ^2^Monash Newborn, Monash Children's Hospital, Melbourne, VIC, Australia; ^3^Department of Obstetrics and Gynaecology, Monash University, Melbourne, VIC, Australia; ^4^Division of Obstetrics and Fetal Medicine, Department of Obstetrics and Gynecology, Erasmus MC, University Medical Center, Rotterdam, Netherlands; ^5^Division of Neonatology, Department of Pediatrics, Leiden University Medical Centre, Leiden, Netherlands

**Keywords:** physiologic-based cord clamping, hypothermia, immediate cord clamping (ICC), delayed cord clamping (DCC), temperature stability, neonate

## Abstract

**Background:** Physiologic-based cord clamping (PBCC) involves deferring umbilical cord clamping until after lung aeration. It is unclear if infant is at risk of becoming hypothermic during PBCC.

**Objectives:** To test if PBCC would maintain core temperature more effectively than immediate cord clamping (ICC).

**Design:** At 0.93 gestation, fetal lambs were surgically exteriorized and instrumented from pregnant ewes under general anesthesia. Prior to the start of the experiment, lambs were thoroughly dried, placed on hot water bottles, and core temperature was continuously monitored using a rectal thermometer. PBCC lambs (*n* = 21), received intermittent positive pressure ventilation (iPPV) for ≥5 min prior to umbilical cord clamping. In ICC lambs (*n* = 23), iPPV commenced within 60 s after umbilical cord clamping. iPPV was provided with heated/humidified gas. Lambs were moved under a radiant warmer after umbilical cord clamping. Additional warmth was provided using a plastic overlay, hairdryer, and extra water bottles, as needed. Two-way mixed and repeated measures one-way ANOVAs were used to compare temperature changes between and within a single group, respectively, over time.

**Results:** Basal fetal parameters including core temperature were similar between groups. ICC lambs had a significant reduction in temperature compared to PBCC lambs (*p* < 0.001), evident by 1 min (*p* = 0.002). ICC lambs decreased temperature by 0.51°C (± 0.42) and 0.79°C (± 0.55) at 5 and 10 min respectively (*p* < 0.001). In PBCC lambs, temperature did not significantly change before or after umbilical cord clamping (*p* = 0.4 and *p* = 0.3, respectively).

**Conclusions:** PBCC stabilized core temperature at delivery better than ICC in term lambs. Hypothermia may not be a significant risk during PBCC.

## What is Known About This Topic

- Delayed cord clamping is recommended for all vigorous newborns because of improved hemodynamic stability and placental transfusion.
- Physiologic-based cord clamping is deferring umbilical cord clamping until the non-vigorous newborn has commenced breathing or respiratory support has started and the lung has aerated.
- Concerns of hypothermia are a barrier to implementing both delayed cord clamping and physiologic based cord clamping.

## What This Study Adds

- Physiological-based cord clamping maintained core temperature stability compared to immediate cord clamping in near-term, newborn lambs.
- Immediate cord clamping resulted in a significant decrease in core temperature in anesthetized, near-term lambs, despite extensive efforts to maintain euthermia in all subjects.
- PBCC utilizes the birthing mother as a potential source of heat and may lead to improved temperature stability in non-vigorous term newborns at birth.

## Introduction

Physiologic-based cord clamping (PBCC) involves deferring umbilical cord clamping until after the newborn has commenced breathing or respiratory support has started and the lung has aerated. However, it is not clear whether infants are at increased risk of hypothermia during PBCC, particularly since umbilical cord clamping may be deferred for several minutes, delaying the time between birth and when the infant receives heat from the radiant warmer (1–4).

Hypothermia increases the risk of morbidity and mortality in the newborn, especially in resource limited settings and remains a common problem for infants that require NICU admission in resource abundant settings (5–14). In low birthweight infants, every 1 degree in admission temperature increased the risk of mortality by 28% and late on-set sepsis by 11% (15). Interventions to maintain stable temperatures after birth include drying the newborn, plastic wrap, warm towels and hats, humidified and heated gas during respiratory support, and placing the infant under a radiant warmer (12–14). Deferred umbilical cord clamping in vigorous preterm and term infants is currently recommended for at least 60 s (16, 17). Randomized trials comparing deferred umbilical cord clamping to immediate cord clamping (ICC) have not shown increased risk of hypothermia (18–21). However, these studies enrolled vigorous infants. Delays before placing the infant on the warming bed were brief. There is a growing body of evidence that providing respiratory support prior to umbilical cord clamping is feasible and may be beneficial for even the most vulnerable and non-vigorous newborns (1–4, 22, 23).

Depending on gestational age, before birth a large percentage of fetal cardiac output (30-50%) perfuses the placenta and ~30% of fetal blood volume is in the placenta at any moment in time. Because of the high perfusion rate and the fact that the placenta remains *in utero* during PBCC, it is possible that maternal body heat is a potentially overlooked source of heat for the newborn (24–26). That is, the infant's blood may undergo re-heating to maternal core body temperature as it perfuses the placenta, prior to returning to the newborn. We aimed to explore core temperature changes in near-term lambs who received PBCC or ICC. We hypothesized that PBCC would improve temperature stability and reduce hypothermia in near-term lambs compared to lambs undergoing ICC.

## Materials and Methods

All experimental procedures were performed in accordance with the National Health and Medical Research Council Code of Practice for the Care and Use of Animals for Scientific Purposes and were approved by Monash University “MMCA” Ethics committee. This is a secondary analysis of core temperature stability using data from primary experiments that compared cardiopulmonary changes in lambs that were managed with ICC vs. PBCC. We reviewed electronic experimental records performed at our lab between 2015 and 2018 for eligible lambs to include in the analysis. Eligible lambs were delivered at ~138 days, had a baseline temperature ≥36.5°C, baseline fetal pH ≥7.1, and had a rectal temperature probe that recorded continuous information. Baseline temperature was defined as the average temperature in the 30 s prior to the start of the experiment. Lambs in the PBCC arm were required to have ≥5 min of ventilation, an increase in pulmonary blood flow, and a pattern of left to right shunting seen in the pulmonary artery flow probe prior to umbilical cord clamping. Lambs were excluded if they were excluded from the analysis in the primary experiments because of clinical instability. The lambs analyzed in this study were selected from previously conducted experiments investigating physiologic changes during birth comparing PBCC to ICC in near-term lambs with congenital diaphragmatic hernia (CDH), lung hypoplasia, and healthy control lambs or lambs that had sham procedures and did not have cardiorespiratory issues (27–32). Investigators aimed to maintain normal temperatures in all lambs (≥38°C) in order to avoid a confounding variable to the measured outcomes of the primary experiments. A temperature of ≤ 37°C prompted investigators to apply additional warming measures, like the use of a hairdryer, replacing the dry towels, and further drying.

### Surgical Preparation and Instrumentation

Pregnant border-leicester ewes underwent cesarean section at 138 ± 1 days' gestation (approximately equivalent to 36 weeks' gestation in humans, full term is ~149 days) under general anesthesia as previously described (27–30, 32). Near-term fetal lambs were exposed by hysterotomy. Polyvinyl catheters (20 gauge) were inserted into the left fetal carotid artery and jugular vein. The fetal trachea was intubated with a 4–4.5 mm cuffed endotracheal tube and lung liquid was passively drained prior to commencing intermittent positive pressure ventilation (iPPV). An ultrasonic flow probe (Transonic Systems, Ithaca, USA) was placed around the left main pulmonary artery to measure pulmonary blood flow. A temperature probe (Large Animal Rectal Probe, ADInstruments, NSW, Australia) was inserted into the rectum for continuous core temperature monitoring. All data were digitally recorded throughout the study and visible on a large, high-definition screen for continuous monitoring by the investigators during the experiment (Labchart, ADInstruments, NSW, Australia). All experiments were conducted in a single, climate-controlled room with an ambient temperature of 20°C.

### Experimental Intervention, Ventilation, and Monitoring

In ICC lambs, the experiment commenced with umbilical cord clamping, whereas in the PBCC lambs the experiment commenced with ventilation onset. Near-term lambs randomized to the ICC group received mechanical iPPV within 60 s of umbilical cord clamping. In lambs randomized to the PBCC group, mechanical iPPV commenced >5 min before umbilical cord clamping while the lambs remained on the ewe's abdomen. Tidal volume targets and increases in pulmonary blood flow were used as indicators for lung aeration and transition of cardiopulmonary transition from fetal to neonatal life. These indicators determined when the investigators clamping the umbilical cord in PBCC lambs. Efforts to maintain euthermia began during the instrumentation process and continued throughout the experiment by continuously monitoring core body temperatures. In both groups of lambs, exposure to the *ex-utero* environment greatly increased the potential for heat loss, which was minimized by thoroughly drying the lambs, replacing wet towels with dry towels, placing lambs on hot water bottles, and covering exposed areas with plastic wrap. Additional heat was provided using a hairdryer if temperature dropped below 37°C. Lambs in both groups were transferred to the infant warming bed as soon as possible after umbilical cord clamping. The warming bed had a fresh hot water bottle and an overhead radiant warmer set on 100% manual heat (Cosy Cot Infant Warmer, Fisher and Paykel, Auckland, New Zealand). If core temperature rose above 39°C, the radiant warmer was adjusted and the plastic wrap and/or hot water bottles removed as required.

In all subjects, ventilation began with a sustained inflation (30 cmH_2_O for 30 s) delivered via a T-piece positive pressure inflation device (Neopuff, Fisher and Paykel Healthcare, Panmure, Auckland, NZ), followed by volume-guaranteed mechanical iPPV at 4–7 ml/kg with a positive end-expiratory pressure of 5 cmH_2_O, rate of 60 inflations per minute, inspiratory time of 0.5 s and fraction of inspired oxygen (FiO_2_) of 0.21 (Babylog 8000 Plus ventilator, Drager, Lubeck, Germany). The sustained inflation was dry, unheated air. Once on the ventilator, the gas was humidified and heated to 37°C (MR850, Fisher & Paykel, Auckland, New Zealand) (12). Ventilation and FiO_2_ adjustments were made as needed to maintain pre-specified targets per experimental protocols. Arterial blood gas samples were collected at baseline, prior to the start of the experiment, then every 5 min using an ABL700 Blood Gas Analyser (Radiometer, Copenhagen, Denmark). All lambs were ventilated for at least 30 min. After umbilical cord clampig, all lambs began an infusion of Alfaxan (5–15 mg/kg/h, Jurox, Rutherford, Australia) in 5% dextrose to maintain sedation during the experiment. Ewes were administered a lethal dose of sodium pentobarbitone (100 mg/kg intraveneously, Jurox, Rutherdord, Australia) following umbilical cord clamping. Lambs were administered a lethal dose of sodium pentobarbitone (100 mg/kg) at the end of the experiment.

### Analysis and Statistics

Baseline and fetal data were analyzed using paired Student's *t*-test for parametric data and Mann Whitney *U* test for non-parametric data. Two-way mixed ANOVA with Games-Howell *post-hoc* analysis was performed to compare continuous variables between groups. One-way repeated measures ANOVA with Bonferroni correction for multiple comparisons was used for parametric data to analyze the changes in continuous variables over time within each group. Normal data are presented as mean ± standard deviation and non-normative data is presented as median (interquartile range). SPSS (Version 25, IBM, Armonk, USA) was used for all statistical calculations. Statistical significance was accepted as *p* < 0.05.

## Results

### Subject Selection, Baseline Fetal Characteristics, and Experimental Conditions

**Figure 1:**
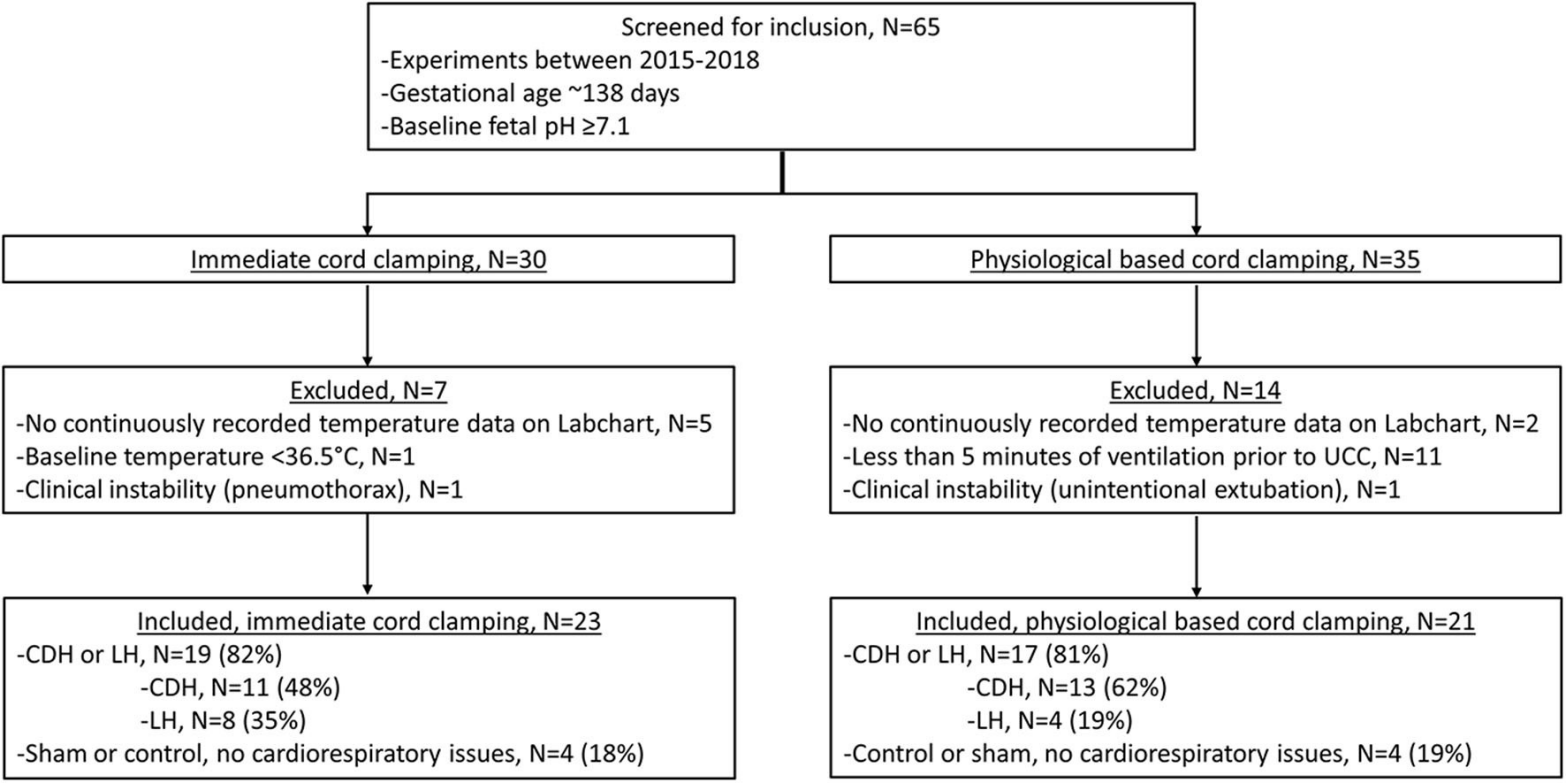
Criteria for inclusion and exclusion, CDH, congenital diaphragmatic hernia; LH, lung hypoplasia.

We screened 65 lambs for eligibility; 30 lambs received ICC and 35 lamb received PBCC (Figure 1). Seven ICC lambs and 14 PBCC lambs were excluded. We included 23 lambs that received ICC and 21 lambs that received PBCC. Fetal baseline characteristics, including basal temperature, weight, gender, and blood gas status were similar between groups (Table 1). Ventilation began at 15 (IQR 6-57) s after umbilical cord clamping in ICC lambs, whereas, in PBCC lambs, ventilation began 617 (IQR 430-634) s before umbilical cord clamping. ICC lambs were moved to the warming bed significantly sooner than PBCC lambs (ICC: 300 (IQR 30-490) s vs. PBCC: 694 (650-739) s, *p* < 0.001). Pulmonary blood flow increased significantly during the first 10 min of ventilation in both groups (*p* < 0.001 for ICC and PBCC) but there was no difference in pulmonary blood flow between groups (*p* = 0.5). There were no significant differences in pH and CO_2_ in the first 10 min after ventilation onset between groups.

**Table 1:** Baseline characteristics presented in mean and standard deviation or median and interquartile range (25 to 75%), kg, kilograms; temp, temperature; s, seconds; C°, degrees Celsius; CO_2_, carbon dioxide; mmHg, millimeters of mercury, *denotes significant difference (p <0.05).

	**Immediate cord clamping, *N* = 23**	**Physiologic based cord clamping, *N* = 21**	***p*-value**
Weight, kg	4.29 ± 0.7	4.17 ± 0.6	0.56
Gender, % Male	63%	61%	0.93
Fetal temp, C°	37.8 ± 0.7	38.1 ± 0.6	0.14
Fetal pH	7.24 ± 0.06	7.21 ± 0.08	0.11
Fetal CO_2_, mmHg	64 ± 7	67 ± 10	0.32
Umbilical cord clamp, s	0 ± 0	617 (430-634)	NA
Umbilical cord clamp to ventilation, s	15 (6–57)	NA	NA
Time to place lamb under radiant warmer, s*	300 (30–490)	694 (650-739)	<0.001

### Temperature Changes Following Ventilation Onset

**Figure 2:**
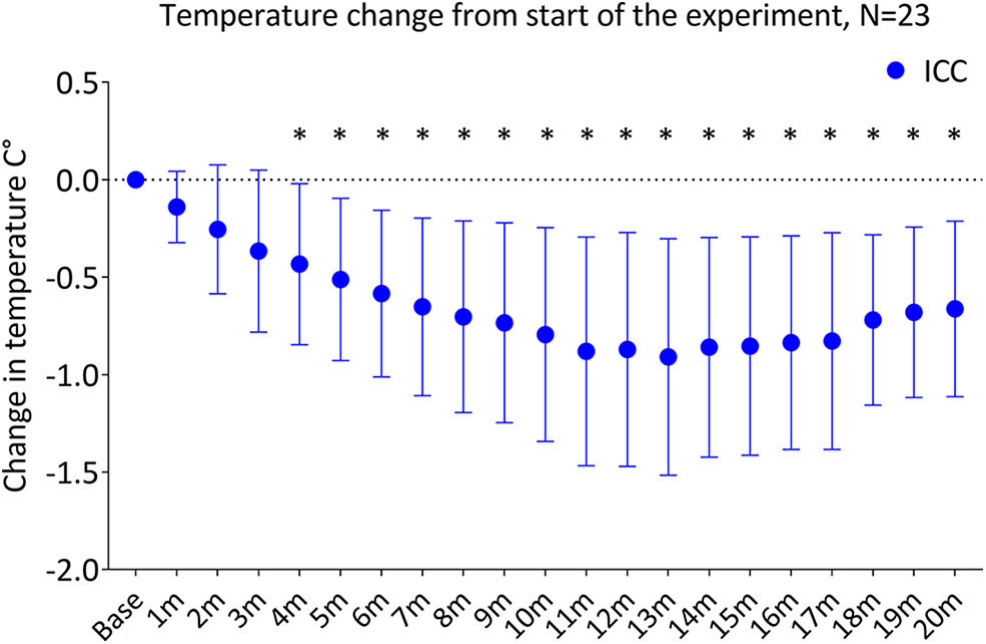
Change in temperature from the start of the experiment in lambs receiving immediate cord clamping (ICC) displayed in mean ± SD using one-way repeated measures ANOVA with Bonferroni correction, m, minutes, *denotes significant difference (*p* < 0.05) in temperature from baseline.

**Figure 3:**
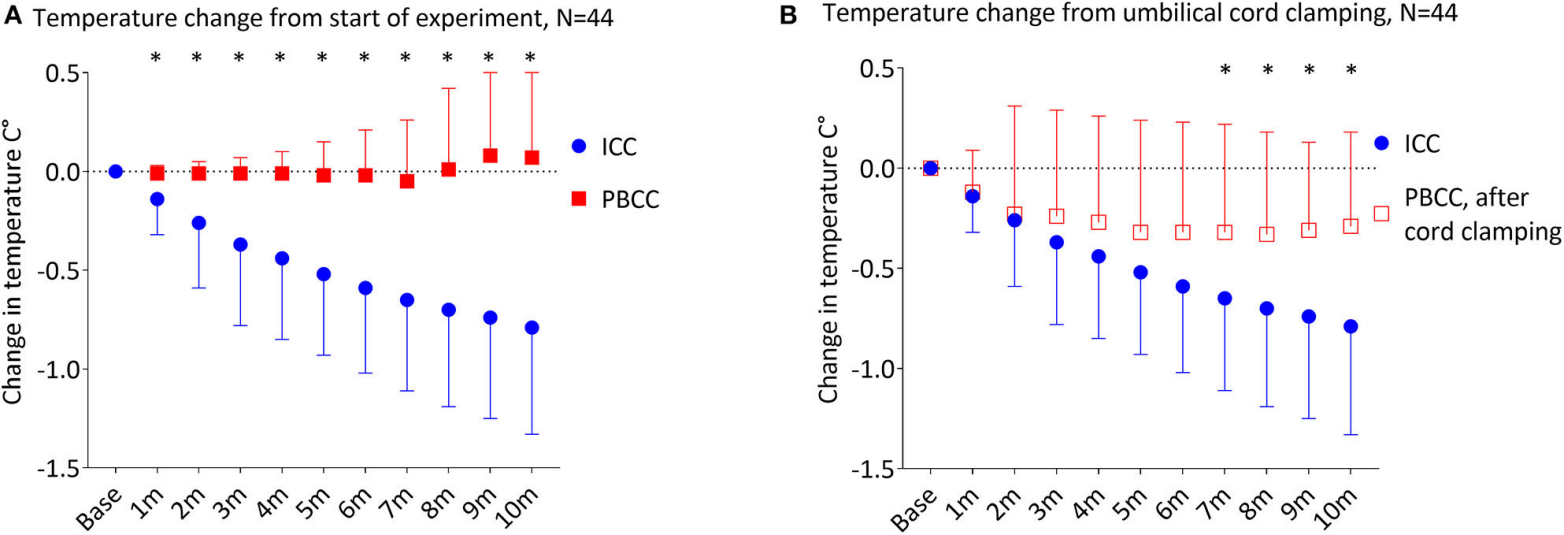
**(A)** Change in temperature from the start of the experiment in lambs receiving immediate cord clamping (ICC) vs. physiologic-based cord clamping (PBCC) displayed in mean ± SD using two-way mixed ANOVA with Games-Howell *post-hoc* analysis. **(B)** Change in temperature from the time of umbilical cord clamping in lambs receiving ICC vs. PBCC displayed in mean ± SD using two-way mixed ANOVA with Games-Howell *post-hoc* analysis. ICC, immediate cord clamping; PBCC, physiological based cord clamping; m, minutes, *denotes significant difference (*p* < 0.05) in temperature between groups.

Temperature decreased significantly in the ICC group over the first 20 min of the experiment (*p* < 0.001), with a significant decrease from baseline temperature by 4 min (*p* = 0.01). By 5 and 10 min of the experiment, temperature in the ICC lambs decreased by ^−^0.51 ± 0.42°C and ^−^0.79 ± 0.55°C, respectively (Figure 2, Table 2). Mean group temperature in the ICC lambs reached a nadir of ^−^0.95 ± 0.78°C from baseline 13 min after starting the experiment (*p* < 0.001). The average maximum decrease in temperature for individual ICC lambs was ^−^1.1 ± ^−^0.5°C at 12 ± 4 min (*p* < 0.001). By 20 min temperature had stabilized and increased to ^−^0.7 ± 0.5°C from baseline. In comparison, temperature in the PBCC group did not change over time (*p* = 0.4). Compared to ICC lambs, temperature in the PBCC lambs was significantly higher by 1 min (Figure 3A, *p* < 0.001) and remained higher throughout the first 10 min of the experiment (*p* = 0.002).

**Table 2:** Experimental results presented in means and standard deviation or median and interquartile range (25 to 75%), Temp, temperature; min, minutes; C°, degrees Celsius; UCC, umbilical cord clamping; CO_2_, carbon dioxide; mmHg, millimeters of mercury, *denotes significant difference (p <0.05).

	**Immediate cord clamping, *N* = 23**	**Physiologic based cord clamping, *N* = 21**	***p*-value**
Temp change 5 min, C°*	^−^0.43 (^−^0.28-^−^0.78)	0.0 (^−^0.08-0.09)	<0.001
Temp change 10 min, C°*	^−^0.79 ± 0.55	0.07 ± 0.43	<0.001
Temp change 5 min after UCC, C°	^−^0.51 ± 0.42	^−^0.32 ± 0.57	0.2
Temp change 10 min after UCC, C°*	^−^0.79°C ± 0.55	^−^0.29 ± 0.47	<0.001
Temp nadir, C° and time from UCC, min*	^−^0.95 ± 0.78, 13 min	^−^0.34 ± 0.51, 8 min	<0.001
pH at 5 min	7.21 ± 0.11	7.25 ± 0.07	0.11
pH change at 5 min*	^−^0.04 ± 0.1	0.03 ± 0.03	0.003
pH at 10 min	7.22 ± 0.15	7.25 ± 0.08	0.42
pH change at 10 min	^−^0.03 ± 0.14	0.03 ± 0.06	0.12
CO_2_ at 5 min, mmHg	62 ± 19	57 ± 11	0.22
CO_2_ at 10 min, mmHg	63 ± 26	58 ± 12	0.45

### Temperature Changes With Umbilical Cord Clamping

To determine whether the reductions in temperature observed in ICC lambs were driven primarily by timing of cord clamping, we compared changes in temperature upon umbilical cord clamping in both groups (Figure 3B). The ICC group had a significant decrease in temperature compared to the PBCC group (*p* = 0.003) which reached significance 7 min after umbilical cord clamping (*p* = 0.03). While temperature in the PBCC group did decrease after cord clamping, this was not statistically significance (*p* = 0.3). PBCC lambs reached a nadir of ^−^0.34 ± 0.51°C, 8 min after umbilical cord clamping (Table 2).

### Temperature Changes With Early Movement to the Radiant Warmer

**Figure 4:**
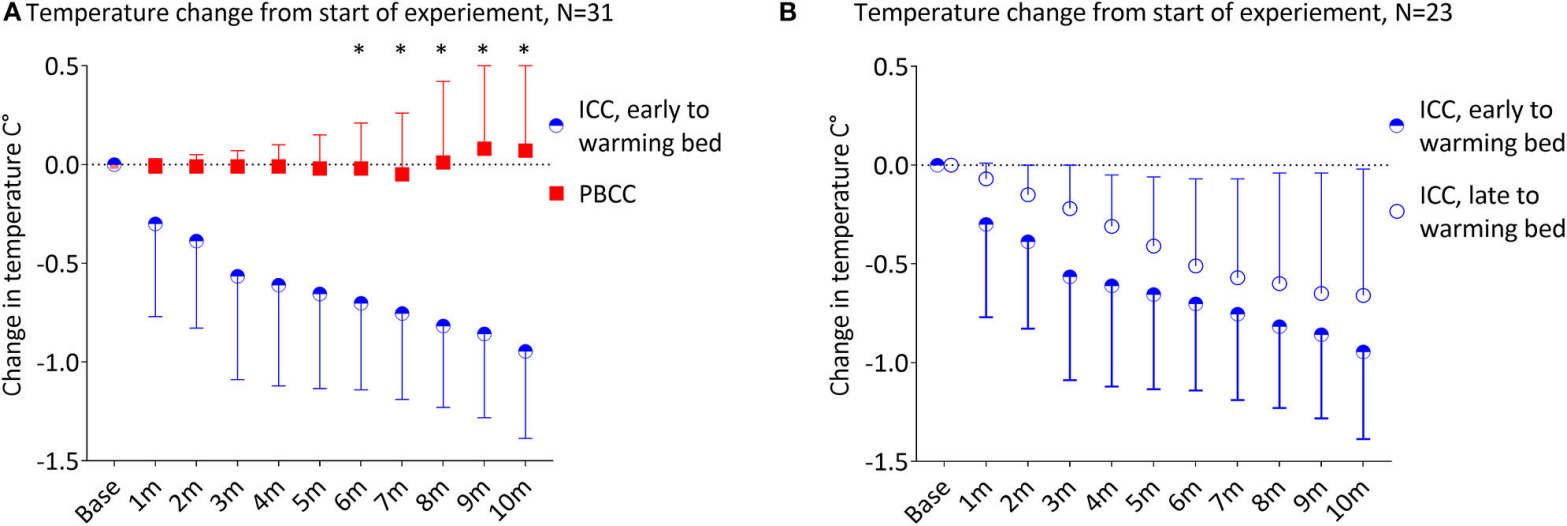
**(A)** Change in temperature from the start of the experiment in lambs that received immediate cord clamping (ICC) and were transferred to the warming bed early (mean 38 ± 20 s after umbilical cord clamping) vs. physiologic-based cord clamping (PBCC) displayed in mean ± SD using two-way mixed ANOVA with Games-Howell *post-hoc* analysis. **(B)** Change in from the start of the experiment in lambs that received ICC and transferred to the warming bed early (mean 38 ± 20 s after umbilical cord clamping) vs. late (mean 412 ± 178 s after umbilical cord clamping) displayed in mean ± SD using two-way mixed ANOVA with Games-Howell *post-hoc* analysis. ICC, immediate cord clamping; PBCC, physiological based cord clamping; m, minutes, *denotes significant difference (*p* < 0.05) in temperature between groups.

Due to differences in protocols of the primary experiments, 10 lambs in the ICC group were moved to the warming bed with a radiant warmer within 60 s of umbilical cord clamping (ICC: 38 ± 20 s vs. PBCC: 683 ± 140 s, *p* < 0.001). Early movement to the warming bed still resulted in a significant decrease in temperature in this subgroup of ICC lambs compared to the PBCC lambs (Figure 4A, *p* < 0.001). The decrease in temperature was significant by 2 min compared to PBCC (*p* = 0.02) and by 5 min (*p* = 0.04) compared to the baseline temperature in this subgroup.

### Temperature Changes in ICC Lambs With Early vs. Late Movement to the Radiant Warmer

We compared the ICC lambs that moved to the warming bed early (*N* = 10) vs. ICC lambs that moved to the warming bed late (*N* = 13, Figure 4B, early warming bed ICC: 38 ± 20 s vs. late warming bed ICC: 412 ± 178 s, *p* < 0.001). There was no significant difference in temperature between the early and late warming bed ICC subgroups during the first 10 min of the experiment (*p* = 0.5). Both ICC subgroups had significant decreases in temperature after umbilical cord clamping (Early warming bed ICC lambs: *p* = 0.006, maximum decrease ^−^0.95 ± 0.14°C at 10 min and late warming bed ICC lambs: *p* = 0.001, with a maximum decrease ^−^0.66 ± 0.16°C at 10 min).

## Discussion

Immediate cord clamping is the current standard of care for the non-vigorous newborn (16). It is thought that this approach allows the infant to receive respiratory support as soon as possible after birth and will minimize the reduction in core body temperature associated with exposure to the extra-uterine environment (33). It has been suggested that PBCC may be associated with an increased risk of hypothermia because the application of radiant heat during PBCC presents a logistical challenge, resulting in delays to placing the infant under the radiant warmer or careful planning to apply radiant heat during deferred cord clamping (1, 3, 4, 34). However, we found that near-term PBCC lambs remained normothermic during PBCC suggesting that the maintenance of the umbilical circulation was able to partially mitigate the increased heat loss caused by fetal externalization. Presumably this is because the placenta remains within the maternal pelvis and that the lamb's blood is continuously being reheated as it flows through the fetal side of the placenta (24–26). Temperature stability may even be superior in PBCC compared to ICC.

During these experiments, we attempted to maintain core temperature of the lambs using both standard (radiant heat warmer after umbilical cord clamping, plastic wrap, and heated-humidified gases during respiratory support) and non-standard (continuous core temperature monitoring, hot air from a hair dryer and hot water bottles) warming techniques because the primary objective of these experiments was to study the differences in cardiovascular changes associated with PBCC vs. ICC (12–14). Despite this, ICC lambs still had significant decreases in body temperature. Anecdotally, we have found that the efforts required to maintain core body temperature were greater following ICC than during PBCC, requiring more non-standard warming techniques. In PBCC lambs, we found that core body temperature was easily maintained by simply drying the lamb and applying one hot water bottle. Following umbilical cord clamping, further drying of the lamb, a hot water bottle, a plastic wrap, overhead radiant warmer and hot air were commonly required.

Umbilical cord clamping in the PBCC group did not reproduce the same decrease in core body temperature as occurred in ICC lambs. A simple explanation could be that with ICC, loss of the “placental heat source” coincides with the time when the lambs are wetter and, therefore, the potential for evaporative heat loss is at its greatest. PBCC lambs may have been dryer when umbilical cord clamping occurred and were transferred to the warming table. Lambs are difficult to effectively dry as they can have well developed levels of fleece, particularly on their extremities. While ICC lambs lost, on average 1°C from baseline at 12 min after umbilical cord clamping, we were eventually able to manage temperature losses and they all had improved temperature stability for the remainder of the experiment.

Warming the infant by allowing its blood to be reheated as it flows through an intact placental circulation may be a superior mechanism of supplying heat to the newborn compared to an external heat source. Similarly, invasive heating techniques that supply warmth directly to the circulatory system in children are extremely efficient. Extracorporeal membrane oxygenation is recommended by some experts in cases of refractory moderate and severe accidental hypothermia, with rewarming times far quicker than reported using external heat sources, but there is a paucity of reports using invasive warming techniques in neonates (35–37).

As PBCC and deferred umbilical cord clamping are approaches that do not rely on sophisticated equipment, they are available in resource limited settings where warming beds are less available, hypothermia is more common, and hypothermia is more associated with increased mortality (7–10). Deferred cord clamping is recommended for all vigorous infants immediately after birth in resource limited settings (38). This experiment demonstrates that PBCC may maintain the core temperature of the non-vigorous infant and augment the effectiveness of skin-to-skin contact to post-resuscitation. Preclinical experiments should also be conducted to determine the effects of ongoing placental circulation on maintaining temperature stability in preterm animals.

Clinical observations have refuted the concept that hypothermia may instigate pulmonary hypertension, but none of these studies were able to comment on whether temperature influences the initial pulmonary vascular dilation immediately after birth (39–41). We found that pulmonary blood flow was unaffected by the modest difference in temperature between groups and increased with ventilation onset as previously reported by our group (42–44). Further studies, with larger differences in core temperature would be needed to confirm consistent with clinical observations and extend our understanding of the effect of temperature on pulmonary blood flow in the immediate newborn period.

## Limitations

The lambs were born under general anesthesia in experimental conditions with mild acidois, which blocks endogenous thermogenic responses, including both shivering and non-shivering thermogenesis. Non-shivering thermogenesis, which is a common mechanism adopted by newborns (including humans), involves oxidation of brown fat that surrounds abdominal organs with high blood flows and so effectively also warms the blood. In this experiment, individual differences in endogenous thermogenic responses between lambs were blunted and we were able to clearly define the role of PBCC in providing superior (compared to ICC) temperature stability after birth. The ambient temperature in the room was lower than the currently recommended range of 23°C to 27°C for preterm infants, but these lambs were closer to 36 weeks in humans and ambient room temperature did not vary between groups (45). Clinicians can be reassured that PBCC likely maintains euthermia in compromised term and near-term newborns, but further consideration is required as to how this relates to very preterm infants.

In 13 ICC lambs, there was a delay in moving the lamb to the radiant warmer because moving the lamb can introduce movement artifacts into continuous physiological recordings during a critical time of the experiment. This delay appeared to have no increased effect on body temperature. Both ICC subgroups (early and late to the warming bed) had significant decreases in core temperature after umbilical cord clamping, compared with the PBCC lambs.

The umbilical cord was clamped for a median of 15 (IQR 4-57) s prior to the initiation of ventilation in ICC lambs. The initial 30 s of ventilation in both groups was provided as a sustained inflation of dry, cold air, followed by ventilation of heated, humidified air. Therefore, the ICC had 60 s without placental circulation and without heated, humidified air, while the PBCC lambs did not. This minute without a source of heat, besides the heated water bottled and dry towels, may have affected our results. However, we felt that the measures to maintain temperature in the ICC group is substantially more than is typically provided to the compromised newborn.

As this was a retrospective analysis, we did not measure maternal core body temperatures, but as baseline fetal body temperatures (Table 1) were similar, we would expect there to be no differences between the groups. In sheep, it is well known that fetal body temperatures closely tracks maternal core temperature and remains ~1°C above maternal core temperatures (46–50). Thus, no difference in baseline fetal body temperatures strongly suggests that maternal core temperatures were also not different.

Physiological-based cord clamping maintained core temperature stability compared to immediate cord clamping. Immediate cord clamping resulted in a significant decrease in core temperature in anesthetized, near-term lambs, despite extensive efforts to maintain euthermia in all subjects. Thus, it appears that PBCC utilizes the birthing mother as a source of heat and may lead to improved temperature stability in term and near-term, non-vigorous newborns who require help breathing at birth.

## Data Availability Statement

The datasets presented in this article are not readily available because data used for this analysis was produced from experiments conducted at the Ritchie Centre. Requests for access to raw data will be considered on request. Requests to access the datasets should be directed to douglas.blank@monashhealth.org

## Ethics Statement

The animal study was reviewed and approved by Monash University MMCA Ethics Committee in accordance with the National Health and Medical Research Council Code of Practice for the Care and Use of Animals for Scientific Purposes.

## Author Contributions

All authors have made significant contributions to the conception and design of the study, acquisition of data, data analysis and interpretation, drafting and revising the manuscript, and final approval. All research was conducted at The Ritchie Centre, Hudson Institute of Medical Research, Monash University in Melbourne, VIC, Australia. DB wrote the 1st draft of the manuscript. DB, KC, AK, KR, SH, and GP analyzed the temperature data. DB, KC, AK, RH, PD, EM, KR, SH, and GP conducted the primary experiments. All authors have reviewed and approve of the submitted version of the manuscript and they take full responsibility for the content.

## Conflict of Interest

The authors declare that the research was conducted in the absence of any commercial or financial relationships that could be construed as a potential conflict of interest.

## Supplementary Material

Supplementary Figure 1 Changes in temperature from the start of the experiment in all lambs from the start of the experiment. ICC, immediate cord clamping; PBCC, physiological based cord clamping; m, minutes.

Supplementary Figure 2 Temperature from the start of the experiment in lambs that received immediate cord clamping (ICC) vs. physiologic-based cord clamping (PBCC) displayed in mean ± SD using two-way mixed ANOVA with Games-Howell *post-hoc* analysis. ICC, immediate cord clamping; PBCC, physiological based cord clamping; m, minutes, ^*^ denotes significant difference (*p* < 0.05) in temperature between groups.
