# Supplementary figures and images for: Physiologic-Based Cord Clamping Maintains Core Temperature vs. Immediate Cord Clamping in Near-Term Lambs
Source: Front Pediatr. 2020 Oct 23;8:584983. doi: 10.3389/fped.2020.584983 (PMC7644840; doi:10.3389/fped.2020.584983)

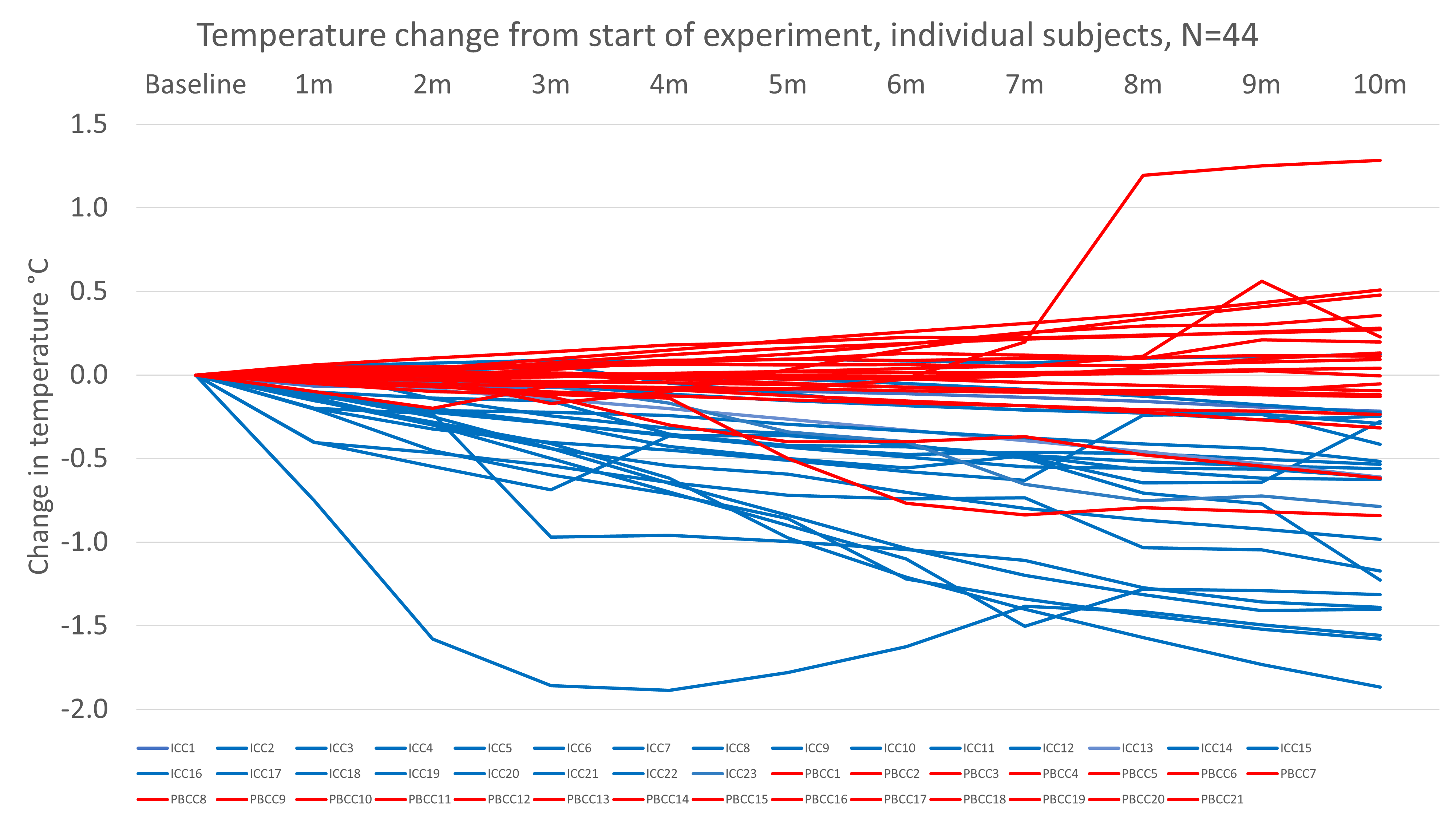

Supplement: Supplementary Figure 1 — Changes in temperature from the start of the experiment in all lambs from the start of the experiment. ICC, immediate cord clamping; PBCC, physiological based cord clamping; m, minutes. [file Image_1.TIF]

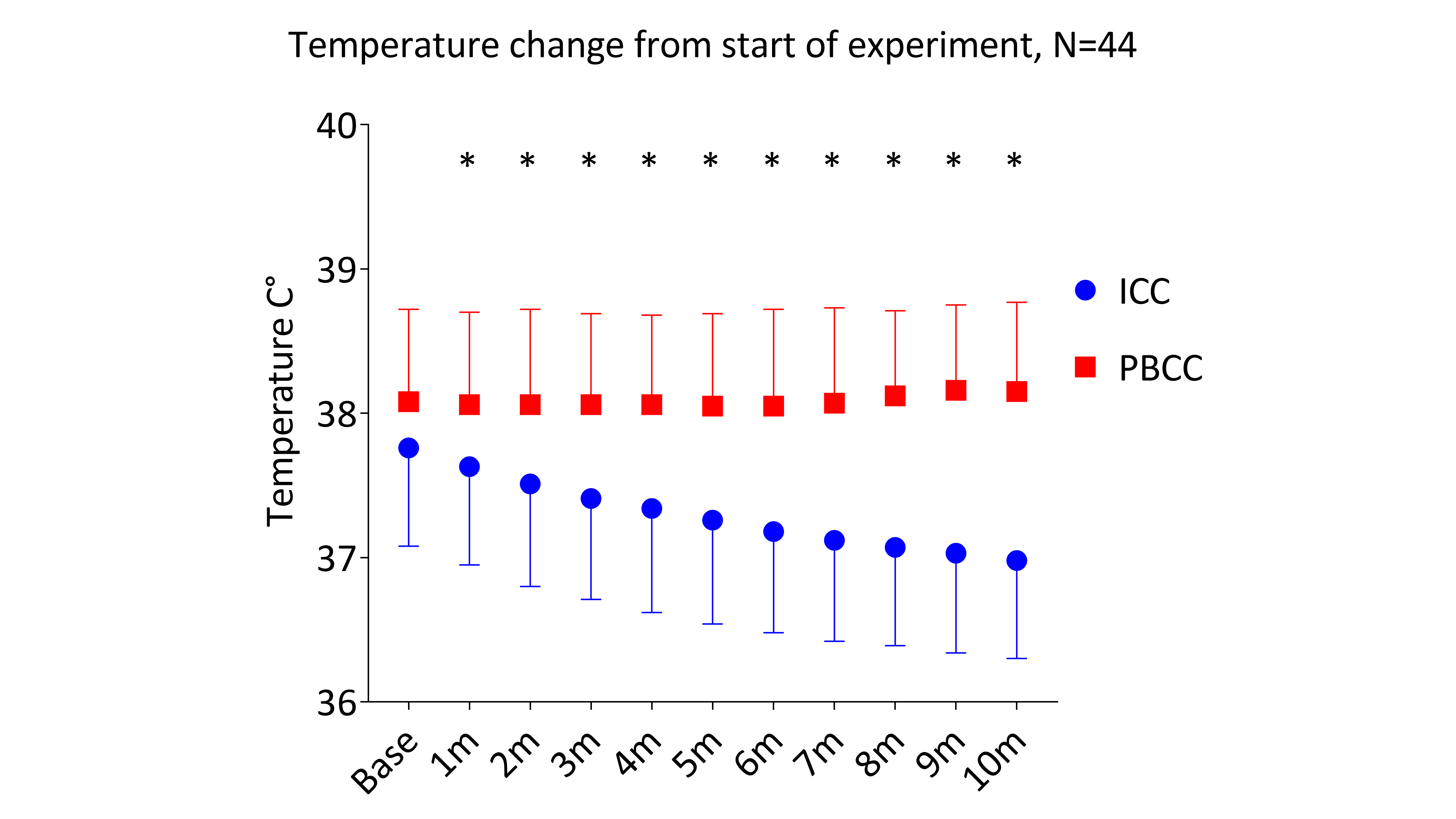

Supplement: Supplementary Figure 2 — Temperature from the start of the experiment in lambs that received immediate cord clamping (ICC) vs. physiologic-based cord clamping (PBCC) displayed in mean ± SD using two-way mixed ANOVA with Games-Howell post-hoc analysis. ICC, immediate cord clamping; PBCC, physiological based cord clamping; m, minutes, * denotes significant difference (p < 0.05) in temperature between groups. [file Image_2.TIF]
